# Gastrointestinal Stromal Tumor of the Rectum: Report of a Case With Long-Term Imatinib Treatment

**DOI:** 10.7759/cureus.74269

**Published:** 2024-11-22

**Authors:** Madhumita Tripathi, Roli Purwar, Richie Sinha, Pooja Singh, Manoj Pandey

**Affiliations:** 1 Surgical Oncology, Institute of Medical Sciences, Banaras Hindu University, Varanasi, IND; 2 Obstetrics and Gynaecology, Institute of Medical Sciences, Banaras Hindu University, Varanasi, IND; 3 Oncological Research, Institute of Medical Sciences, Banaras Hindu University, Varanasi, IND

**Keywords:** gastrointestinal stromal tumor (gist), imatinib mesylate, organ preservation, rectal tumor, tyrosine kinase receptor inhibitors

## Abstract

Gastrointestinal stromal tumors (GIST) are rare in the rectum. These usually present with symptoms produced by compression of pelvic organs or bleeding. Surgery is the treatment of choice, however, at times the surgery can be mutilating and organ preservation may not be possible. In such cases imatinib can be used. We report a case of rectal GIST that presented with urinary obstruction and obstipation treated with long-term imatinib with very good response and relief of symptoms, which prompted the patient to refuse surgery and resulted in organ preservation.

## Introduction

The term gastrointestinal stromal tumor (GIST) was first coined by Mazur et al. in 1983 [1]. GISTs, although a rare condition making up less than 1% of all gastrointestinal tumors, are the commonest mesenchymal tumors of the digestive tract. GISTs express CD117, which can be detected by immunohistochemistry. About 60% of the GISTs occur in the stomach, and around 35% develop in the small intestine. The remaining occur in the rectum, colon, and rarely in the esophagus.

These tumors have spindle-cell or epithelioid histology; KIT (CD117) protein is expressed in 80% of cases, platelet-derived growth factor receptor alpha (PDGFRA) is expressed in 10% and succinate dehydrogenase (SDH) gene in 5-10% [2,3], other infrequent genes like neurofibromatosis 1 (NF1), and ETV6-NTRK3 fusion are also reported [4,5]. Gain-of-function mutations in the KIT proto-oncogene or PDGFRA are linked to their origin and are used to classify GISTs [2]. They result in the constitutive activation of KIT signaling pathway [3]. We report here a case of GIST in the rectum that was treated with long-term imatinib.

## Case presentation

A male in his 50s presented to emergency in May 2019 with acute urinary retention and bowel obstipation for 12 hours. General and abdominal examination was not contributory, and digital rectal examination revealed a hard bulge in anterior rectal wall, mucosa was free. An X-ray abdomen including kidney, ureter, and bladder (KUB) showed a radiopaque shadow in right pelvic area. Ultrasonography (USG) suggested significant residual urine with enlarged prostate. Foleys catheterization was done to relieve urinary retention and patient passed stool after enema. Patient had no known comorbidity, no chronic drug usage, no history of melena, rectal bleed, or hematuria. There was only the history of recent onset increased frequency of micturition. A contrast-enhanced computed tomography (CECT) abdomen and pelvis revealed an 88x87x90 mm mass in pelvic cavity, displacing the rectum on the right side.

**Figure 1:**
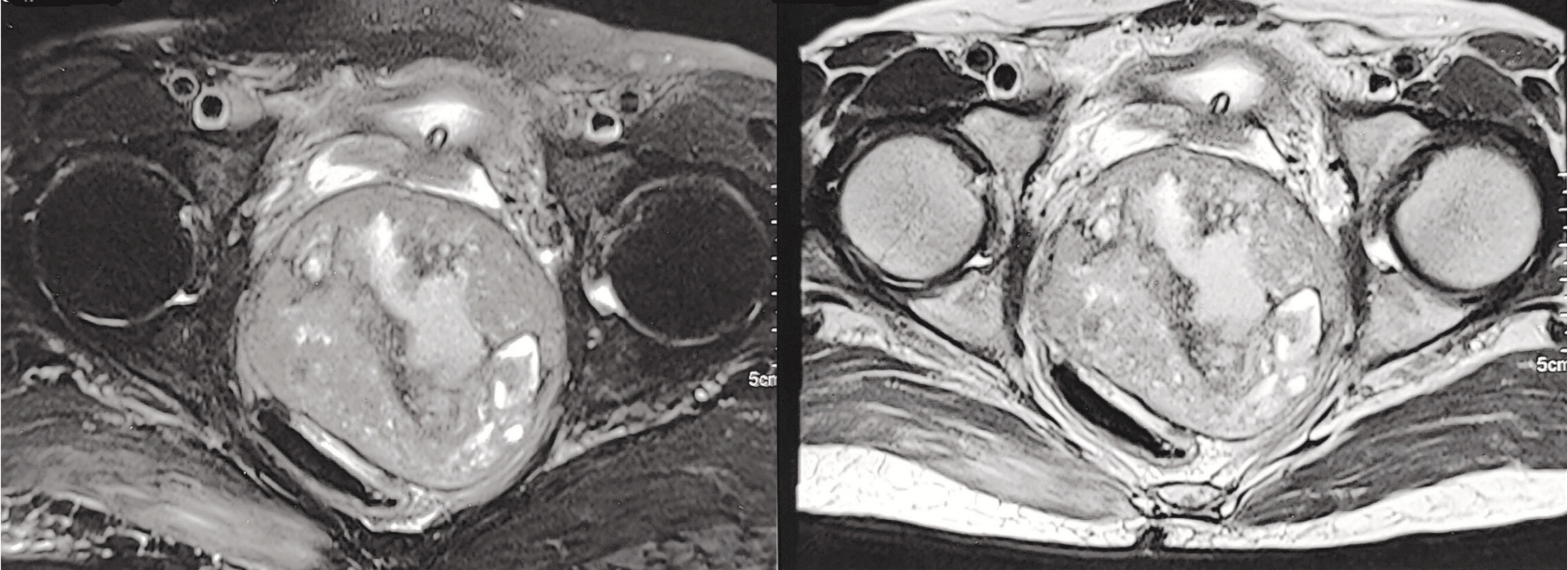
MRI pelvis showing solid cystic mass (88x87x90 mm) lesion in lesser pelvic cavity that was hyperintense in T2 weighted images in T1 weighted images, hyperintense area- compressing rectum towards right with narrowing of its lumen with focal loss of intervening fat planes is seen

MRI pelvis suggested a well-demarcated solid cystic mass (88x87x90 mm) lesion in lesser pelvic cavity that was T2 weighted hyperintense with internal T1 hyperintense area, compressing rectum towards right with narrowing of its lumen with focal loss of intervening fat planes. Mass was compressing prostate anteriorly and inferiorly extending up to anorectal junction with mass effect on bulbo-membranous urethra. Laterally the mass was seen extending to pelvic side wall without obvious infiltration of the pelvic muscles (Figure 1).

**Figure 2:**
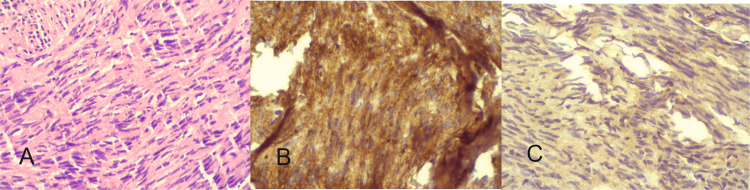
Photomicrograph showing A) hematoxylin & eosin staining showing spindle cell neoplasm (H&E x40) B) intense CD117 positivity (CD117x40) and C) Dog 1 positivity (Dog1x40)

Image-guided Trucut® (Travenol, Deerfield, IL, USA) multicore biopsy suggested spindle cell neoplasm. Immunohistochemistry (IHC) showed CD99+, CD117+, DOG1+, and Vimentin+. KI 67 was 2-3% (Figure 2).

Positron emission tomography (PET) CT showed metabolically active peripherally heterogenous soft tissue density lesion in pelvis in close relation to rectum with compression and loss of fat plane with seminal vesicle and prostate gland. No other distant metastasis was observed.

A diagnosis of locally advanced rectal GIST with multiorgan infiltration was made. Pelvic exenteration was planned and the procedure was explained to the patient who refused surgery. After refusal by patient, the case was discussed in a multidisciplinary meeting and it was decided to start him on imatinib 400 mg once daily and review after one month.

**Figure 3:**
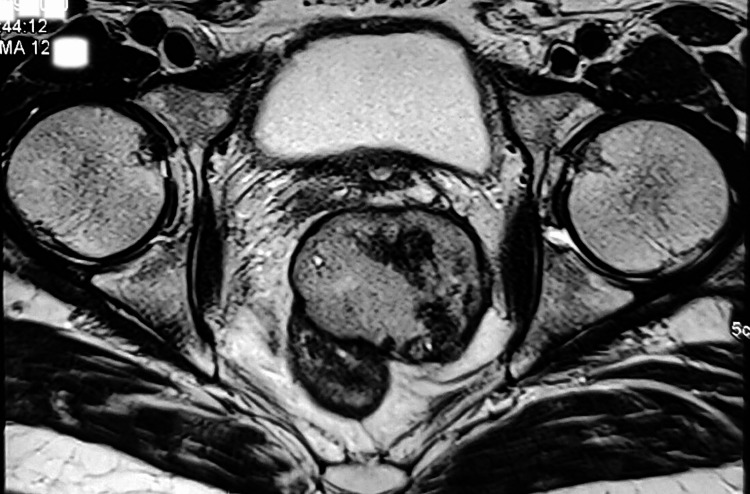
MRI scan on the first follow-up showing significant reduction in size

Patient did not turn up for his scheduled follow-up and reported to outpatient after nine months in February 2020. He had significant improvement with respect to bowel and bladder symptoms. Repeat MRI was done which showed the mass had significantly decreased to 60x40x60 mm, compressing rectum towards right without any luminal narrowing. Mass lesion appeared to be confined within mesorectal space. Caudally, lesion was seen extending up to the level of anorectal junction without significant mass effect on urethra. Laterally the lesion was limited by levator ani muscle without infiltration of lateral pelvic wall (Figure 3).

With this good response and localized disease in mesorectal space, patient was advised to undergo excision of the mass the possibility of losing the sphincter was explained and he again refused the surgery. Patient was advised to continue oral Imatinib at the same dose with a three-monthly follow-up.

**Figure 4:**
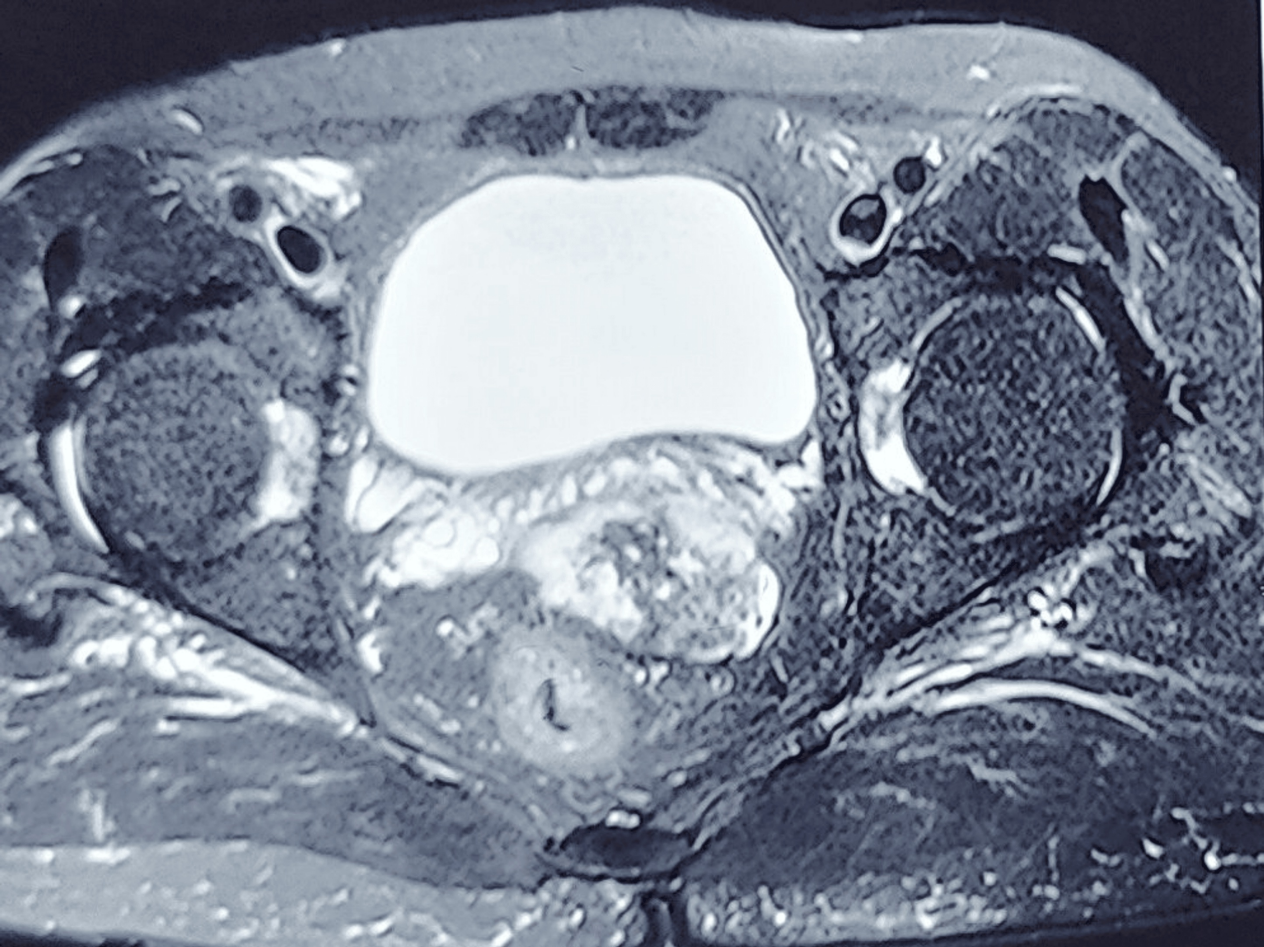
MRI scan on last follow-up showing more than 50% reduction in size and no sign of extra rectal involvement, however; the mesorectum is involved and tumor abuts the seminal vesicle and prostate.

**Figure 5:**
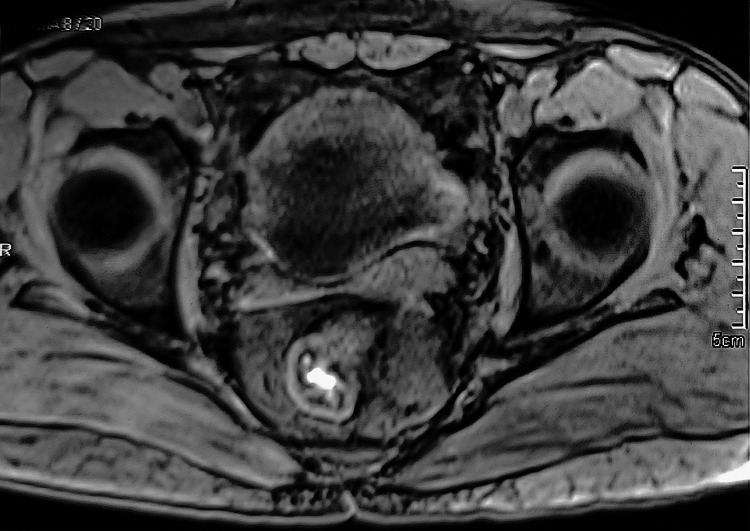
T2 weighted MRI image showing minimal residual disease

Patient again defaulted and did not come for follow-up till August 2022. He had continued taking imatinib and was asymptomatic to date. MRI examination revealed a lesion of 3.5x4.9 cm in size arising from anterior wall of rectum with area of hemorrhage inside the tumor. The mass was abutting the prostate and seminal vesicle (Figure 4). Patient was again counselled for surgery and he again refused, and was advised to continue imatinib. His last follow-up was in October 2024 and repeat MRI showed a residual lesion of 2 cm with no systemic metastasis (Figure 5).

He had continued taking imatinib without default. In view of continued regression in size and absence of new lesions he was advised to continue on imatinib and six monthly follow-up as he is still not willing for surgery.

## Discussion

GISTs in the rectum are rare and constitute approximately 0.1% of all rectal neoplasms [6]. The global data on incidence and prevalence of rectal GIST is not available, however, it is probably the third most common site, constituting up to 5% of all GISTs [7].

Confinement within the bony pelvis makes the treatment of rectal GIST challenging. Historically, rectal GISTs were treated with radical surgeries like abdominoperineal resection (APR) or total pelvic exenteration, if there was extension beyond rectum. GI sarcomas are poorly responsive to chemotherapy leading to higher resistance to chemotherapy [8,9].

Management of GISTs has undergone a paradigm shift after the discovery of systemic therapies in the form of small molecules, the inhibitors of the receptor tyrosine kinases (TKI) that target the mutational activation of KIT or PDGFRA. Imatinib was the first prototype, initially approved for chronic myeloid leukemia (CML) [10]. Later it became clearly evident that imatinib induces clinical benefit in GISTs as well by blocking signaling via the KIT or PDGFRA pathway due to its binding to the ATP-binding pocket that is required for phosphorylation and activation of the tyrosine kinase receptors [11].

In the general population among the patients with GIST, the prevalence of major mutations in KIT is around 75%, and reported to be higher in rectal GIST, mainly occurring in exon 11 [12].

Patients with locally advanced GIST preoperatively treated with TKI’s show considerable response with downstaging of disease and increasing operability in these patients, hence, the neoadjuvant use is on the rise, leading to better organ preservations and longer survival [10]. Recent clinical guidelines recommend that TKI treatment in patients with advanced GIST should be continued until the tumor progression as measured by Response Evaluation Criteria in Solid Tumors (RECIST 1.1) criteria [13].

GISTs are usually seen as extraluminal tumors with heterogeneous enhancement, and intravenous contrast due to high vascularization [14], and contrast-enhanced computed tomography (CT) is the standard imaging method. However, anatomical delineation and detection of adjacent organ involvement are better seen in MRI [15]. FDG‐PET evaluation before treatment is required for baseline assessment but with caution as 20% of GIST do not have abnormal FDG uptake [16]. In this case report, we observed the clinical and radiological response of the rectal GIST using single 400 mg dose of imatinib mesylate daily and used the standardized RECIST 1.1 criteria for response evaluation to neoadjuvant therapy. RECIST 1.1 has allowed for significant improvements in increasing reproducibility and confidence in results [17]. In our case we documented a partial response within six months of therapy with significant symptomatic improvement. Since there was no drug resistance to imatinib therapy, analysis for KIT gene mutation was not done.

The most common adverse events on long-term use of imatinib are edema (63.2%), fatigue (52.6%), skin rash (21.1%), and diarrhea (15.8%) [18]. However, the standard duration of neoadjuvant imatinib therapy before any definitive surgery is not yet defined in literature. The best time for surgery is the point of best response before the development of secondary resistance. In the earlier studies the median time to response is three months, which reaches a plateau at six months [19]. However, our patient has shown a longer response that continued for over five years after starting imatinib without any toxicity. 

## Conclusions

Rectal GISTs are rare tumours, but should be kept in mind as a differential diagnosis when a rectal mass is detected. Diagnostic workup is similar to any other type of rectal neoplasia. Preoperative biopsy and IHC are necessary to confirm the diagnosis and metastatic screening is necessary before starting neoadjuvant or palliative imatinib therapy.
